# Fabrication and Characteristics of ZnO/OAD-InN/PbPc Hybrid Solar Cells Prepared by Oblique-Angle Deposition

**DOI:** 10.3390/molecules17089496

**Published:** 2012-08-08

**Authors:** Cheng-Chiang Chen, Lung-Chien Chen

**Affiliations:** Department of Electro-optical Engineering, National Taipei University of Technology, 1, Section 3, Chung-Hsiao E. Rd., Taipei 106, Taiwan

**Keywords:** indium nitride, zinc oxide, hybrid solar cells, lead phthalocyanine

## Abstract

In this work, lead phthalocyanine (PbPc) and ZnO/InN inorganic semiconductor films prepared by oblique-angle deposition (OAD) were layered to form heterojunction organic/inorganic hybrid photovoltaic solar cells. Among the available organic materials, phthalocyanines, particularly the non-planar ones such as PbPc, are notable for their absorption in the visible and near infrared regions. The organic/inorganic hybrid solar cells fabricated on ZnO/OAD-InN/PbPc showed short-circuit current density (J_SC_), open-circuit voltage (V_OC_), and power conversion efficiencies (η) of 1.2 mA/cm^2^, 0.6 V and 0.144%, respectively.

## 1. Introduction

Organic/inorganic hybrid photovoltaic solar cells are regarded as one of the future high potential molecular electronic application in photovoltaic (PV) systems owing to their low temperature and easy fabrication [1,2,3,4,5,6]. Among organic materials, lead phthalocyanine (PbPc) is a well-known organic semiconductor, and thin films of PbPc have a wide range of possible applications, including solar cells, gas sensors, and electroluminescence devices, owing to its easy synthesis and strong substantial absorption in the visible and near infrared [7,8,9,10]. A TiO_2_-PbPc heterojunction-based organic/inorganic hybrid photovoltaic solar cell was reported and the efficiency was poor (only 0.046%) [11]. However, InN enables III-nitride optical devices to cover the whole visible spectrum and extend into the infrared region, such that InN is a potential material for optoelectronics applications, including solar cells, to substitute for the TiO_2_ layer [12,13,14,15,16,17]. 

Oblique-angle deposition (OAD) is also a coating growth mechanism. As the substrate tilts at an angle, it causes the growth of a columnar structure films due to the shadowing effect during coating. The columnar structure film exhibits high surface roughness characteristics and intrinsically anisotropic physical properties, such as dichroism, birefringence, and anisotropic resistivity [18,19,20,21,22]. Therefore, these material and optical properties are interesting to solar cell applications due to the light behavior inside the OAD materials. In particular, zinc oxide (ZnO) has shown itself to be an attractive substrate material for InN growth [21,22,23]. In addition, oxidation at the ZnO/InN hetero-interface can reduce electron accumulation in the InN ﬁlms for the fabrication of optical and electronic devices [24]. As described in several articles, ZnO has light emission properties with high exciton binding energy [25], high transparency to visible light [26], and high electric conductivity with doping [27]. 

Therefore, this study demonstrated the optical and electronic properties of PbPc. The optical properties of the OAD-InN are also investigated. Finally, this study reported the characteristics of the solar cells with ZnO/OAD-InN/PbPc hybrid structure.

## 2. Experimental

**Figure 1:**
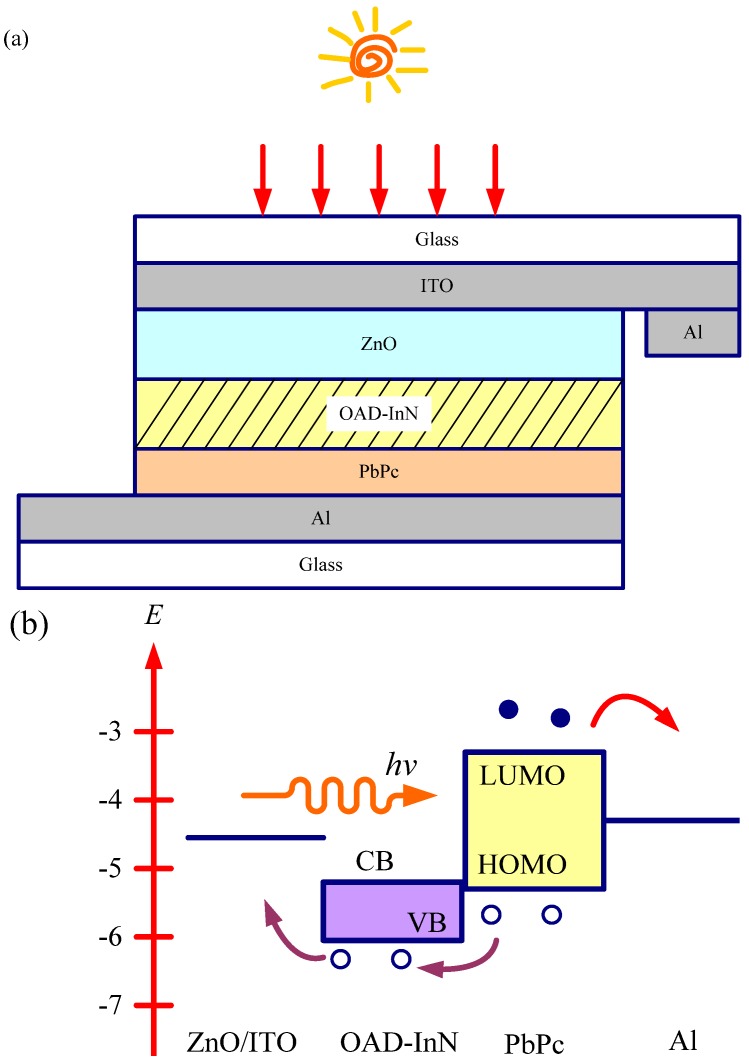
(**a**) Schematic cross-section of the completed structure. (**b**) Energy level diagram and mechanism of photocurrent generation in the solar cells with ZnO/OAD-InN/PbPc structure.

In this study, the organic/inorganic hybrid photovoltaic device has a ZnO/OAD-InN/PbPc structure. Figure 1 shows a schematic cross section of the completed structure. The indium tin oxide (ITO) coated glass substrates (sheet resistance = 15 Ω/□) were cleaned in an ultrasonic bath with de-ionized water, acetone, and methanol for 15 min, respectively. The ZnO thin film was deposited on ITO glass substrates by sputtering and pure ZnO (99.95% in purity) was used as the sputtering target material. Pure argon gas was used as sputtering gas at a flow rate of 40 sccm. Subsequently, OAD-InN on the ZnO film was prepared by radiofrequency magnetron sputtering at an oblique angle using In targets. As for the organic materials, PbPc were dissolved in a chloroform solution (50 mL), in which PbPc (0.3 g), poly(methyl methacrylate) (PMMA) (0.2 g), and I_2_ (0.2 g) were mixed with a magnetic stirrer at room temperature, and were coated on the front side of the OAD-InN/ZnO substrate by spin coating to form an PbPc film with a thickness of around 200 nm. Finally, an aluminum coated glass was employed to contact the PbPc film to construct the whole structure as shown in Figure 1(a). The energy level diagram and mechanism of photocurrent generation in the solar cells with ZnO/OAD-InN/PbPc structure is summarized in Figure 1(b). The resulting morphologies and crystal structures were evaluated by field emission scanning electron microscopy (FESEM) and XRD. The UV–Vis reflectance spectrum was obtained using an optical spectrometer (Hitachi U-4100). Additionally, the current density–voltage (J-V) characteristics were determined using a Keithley 2,420 programmable source meter under irradiation by a 100 mW xenon lamp. Finally, the irradiation power density on the surface of the sample was calibrated as 100 mW/cm^2^. 

## 3. Results and Discussion

**Figure 2:**
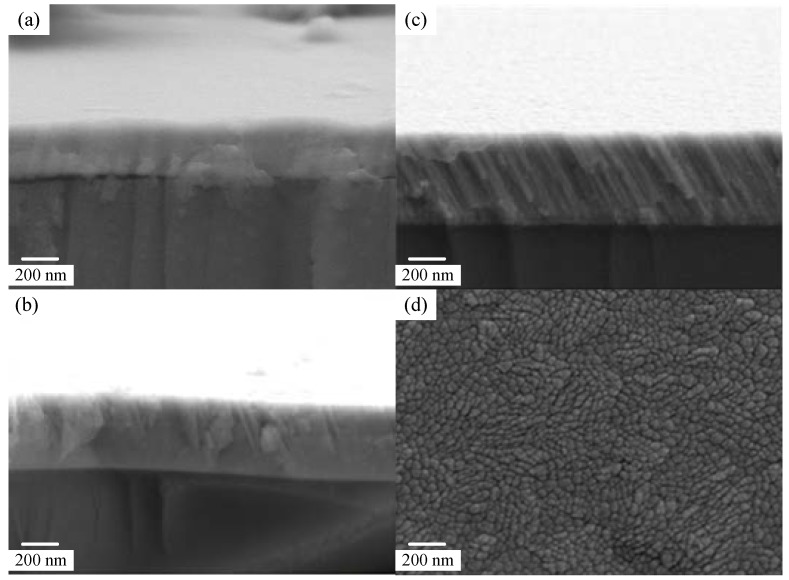
FESEM image of OAD-InN films deposited with variously oblique-angles.

**Figure 3:**
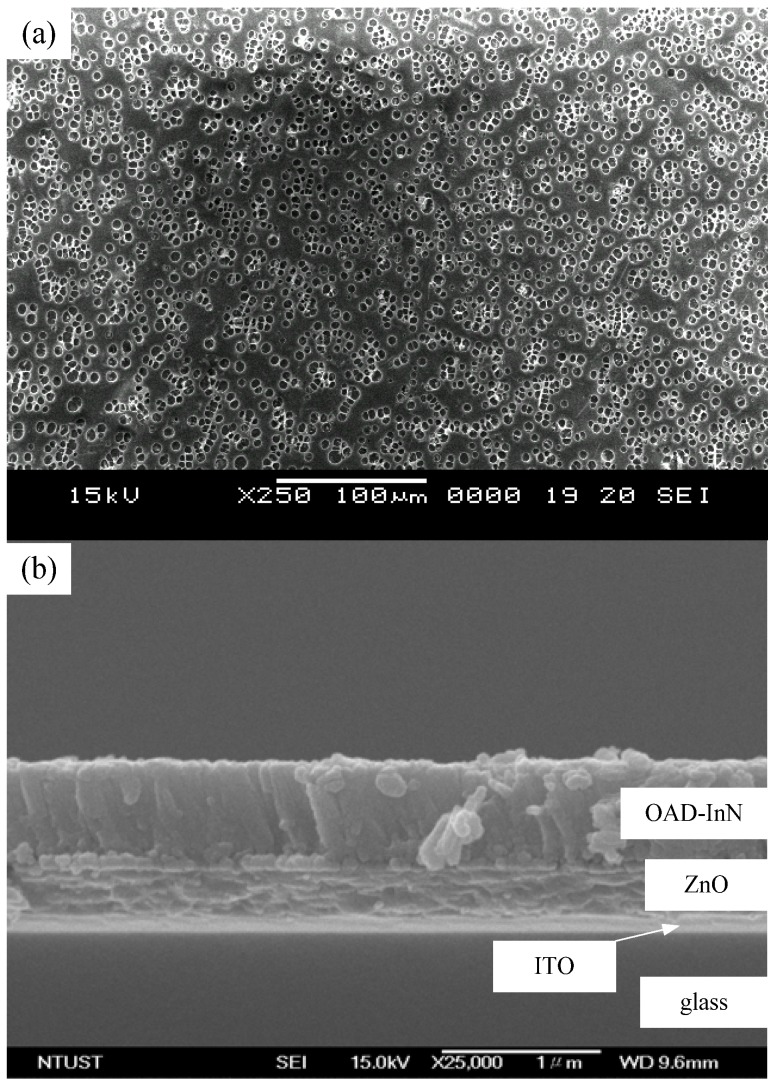
(**a**) Morphology of PbPc layer on glass substrate, and (**b**) cross-sectional FESEM image of OAD-InN/ZnO stack deposited on ITO glass.

Figure 2 shows the top and cross-sectional FESEM images of OAD-InN films that were deposited with various oblique angles. For the normal incidence deposition film, the cross-sectional view shows a featureless structure of the commonly observed columnar structure that is characteristic of a sputter-deposited film. The cross-sectional views of the oblique-angle deposition films at 40° (Figure 2b) and 80° (Figure 2c), show a porous nanocolumn structure. The columnar nanostructure inclination angle increases with the increase in the flux arrival angle caused by the shadow effect [28]. Besides, average pore diameter of the OAD-InN nanocolumnar layer was in the range of 10–30 nm, as shown in Figure 2(d). Figure 3(a) shows the morphology of PbPc layer on a glass substrate with a particle size ranging ~ 3–5 ìm. Figure 3(b) shows the cross-sectional SEM image of OAD-InN/ZnO stack deposited on ITO glass, with a flux arrival angle of 40°.

**Figure 4:**
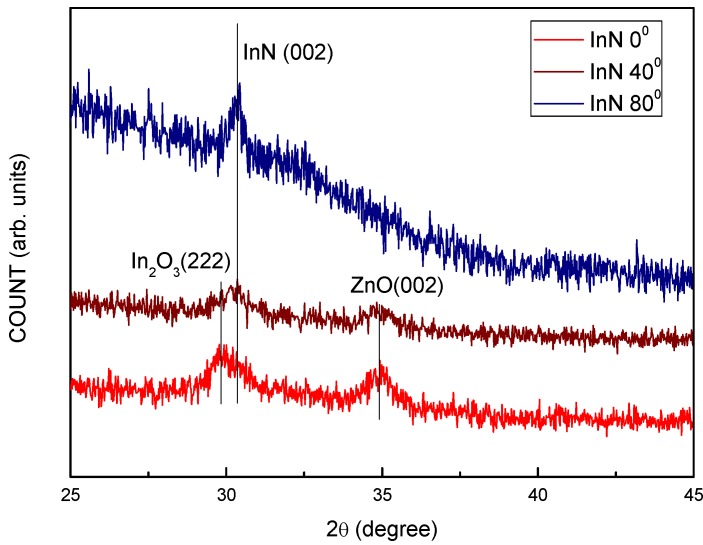
XRD pattern of the OAD-InN films deposited with various oblique angles.

Figure 4 presents a typical XRD pattern of the OAD-InN films deposited at various oblique angles on a ZnO film that has been prepared by magnetron sputtering. Three dominant diffraction peaks [In_2_O_3_(222), InN(002), and ZnO(002)] were observed. When the sputtering oblique angle increases from 0 to 80°, the In_2_O_3_(222) peak vanished, and InN(002) appeared. The oxygen (O) content decreases and the nitrogen (N) content increases in the InN films. This may be attributed to the atomic weight difference between O and N [28]. The origin of oxygen in the InN films may be from the background or residue of the deposition system.

**Figure 5:**
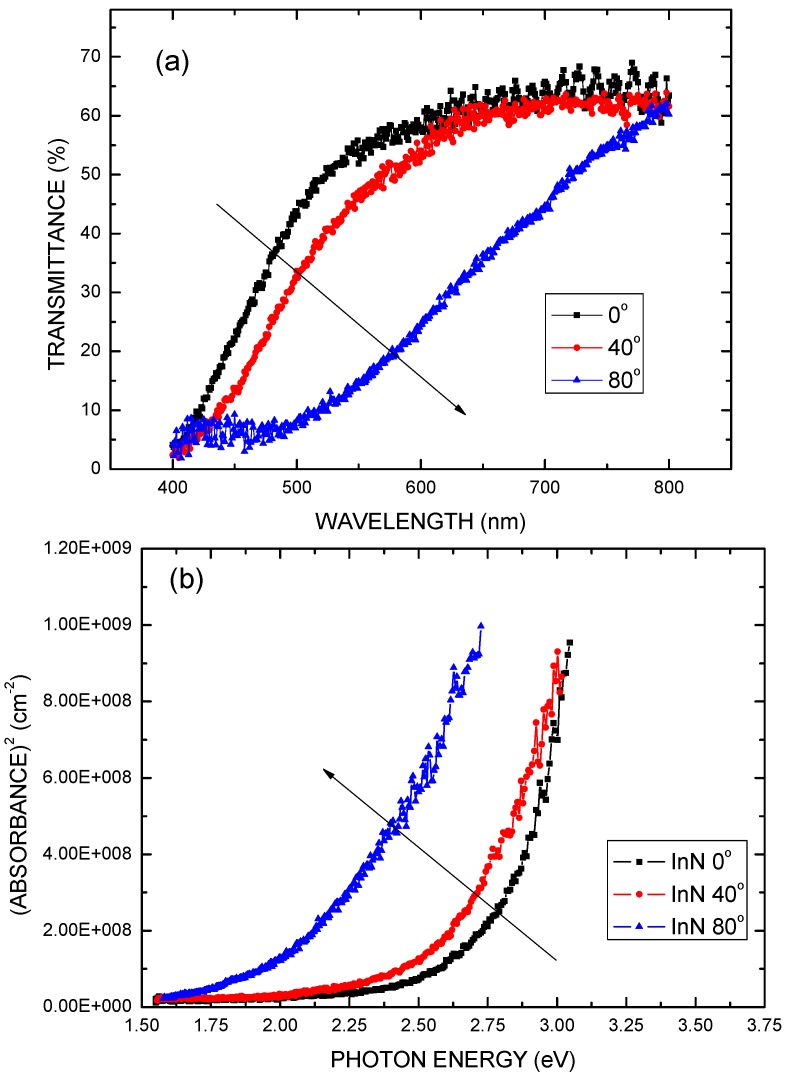
(**a**) Transmittance spectra of OAD-InN films deposited with various oblique-angles. (**b**) Relationship between absorbance squared and photon energy at room temperature for OAD-InN films.

Figure 5(a) plots the transmittance spectra of the OAD-InN films deposited with various oblique-angles in the ranges 400 to 800 nm, and Figure 5(b) shows the results of the absorption measurements for the OAD-InN films. It can be seen from Figure 5(a) and (b) that as the oblique-angle increases, the absorption edge of spectra demonstrates a red shift. The absorption edge at ~2.0 eV (flux angle = 80°) may be attributed to the Burstein-Moss effect due to an increase of the oxygen content in the OAD-InN film. As shown in Figure 4 and Figure 5(b), the OAD-InN films deposited with a higher flux arrival angle may have low absorption edge caused by less oxygen content so that the Fermi level shifts to a higher position from the bottom of the conduction band. 

**Figure 6:**
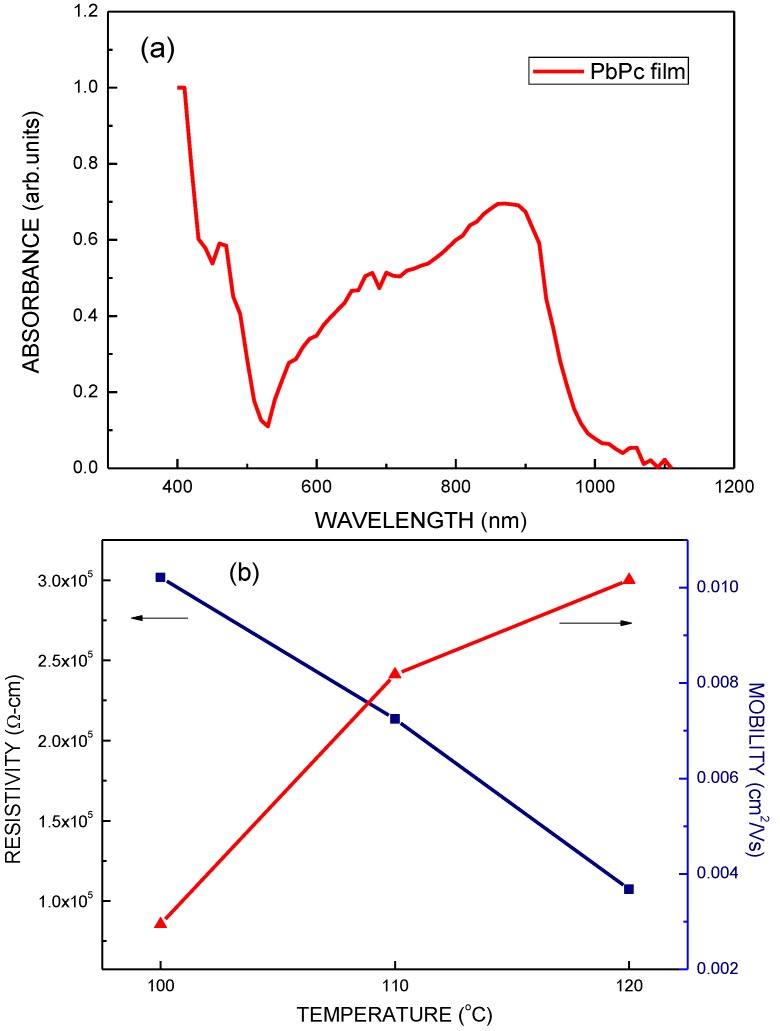
(**a**) Absorbance of PbPc film. (**b**) The relationship between the annealing temperature and the resistivity and mobility of PbPc.

Figure 6(a) shows the absorbance of PbPc film. The PbPc film has a good absorption in the visible and near infrared range. Therefore, in this study, the PbPc film was suitable for the absorption layer of the cell. Figure 6(b) plots the relationship between the annealing temperature and the resistivity and mobility of PbPc. The resistivity decreases and the mobility increases as the annealing temperature increases. The mobility is increased by the PbPc film's annealing. This may be attributed to the charge transport occurs by hopping between adjacent sites through the disordered regions in the PbPc film after annealing treatment [29,30].

**Figure 7:**
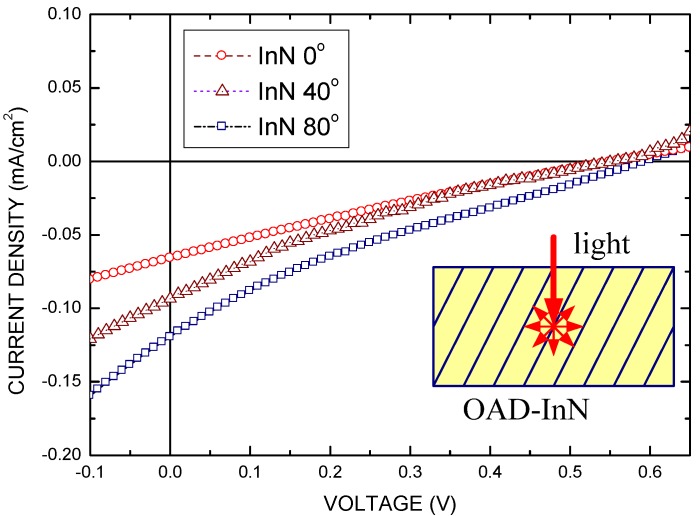
The J-V characteristics of the solar cells with OAD-InN sputtered at various oblique angles. The inset shows the light incident into the OAD-InN film.

Figure 7 shows the *I*-*V* characteristics of the ZnO/OAD-InN/PbPc structure solar cells under illumination, in which the OAD-InNs were deposited at different angles. The cell performance was measured under AM 1.5 illumination with a solar intensity of 100 mW/cm^2^ at 25 °C. The cell has an active area of 0.2 × 0.2 cm^2^ and no antireflective coating. The inset of Figure 7 shows the light incident onto the OAD-InN film. The OAD-InN exhibits better light absorption than that normal InN film because of light scattering in the OAD-InN film. Therefore, the solar cell with OAD-InN film has higher photocurrent than that of the solar cell with the normal InN film. Table 1 lists the characteristic parameters of solar cells with various OAD-InN films. ZnO/OAD-InN/PbPc solar cells, OAD = 80°, exhibited the following static parameters: *J_sc_* of 1.2 mA/cm^2^, and *V_oc_* of 0.6 V. Therefore, the value of the fill factor (*FF*) and the power conversion efficiency (*η*) equals to 0.2 and 0.144%, respectively. Low FF value and conversion efficiency are owing to the high series resistance (~800 Ω, in this study). The series resistance is mainly caused by the resistance of the organic material PbPc and the OAD-InN nanocolumnars. The high series resistance of the OAD-InN nanocolumnars may be caused by the degradation of the carrier transition in the OAD-InN nanocolumnars in the transverse direction.

**Table 1:** The parameters of solar cells with various OAD-InN films.

OAD-InN	V_oc_ (V)	J_sc_ (mA/cm^2^)	FF	*ç* (%)
0°	0.56	0.66	0.225	0.083
40°	0.56	0.94	0.217	0.114
80°	0.60	1.20	0.2	0.144

## 4. Conclusions

In this work, a lead phthalocyanine and ZnO/InN inorganic semiconductor films prepared by oblique-angle deposition (OAD) have been layered to form heterojunction organic/inorganic hybrid photovoltaic solar cells. The organic/inorganic hybrid solar cells fabricated on ZnO/OAD-InN/PbPc shows short-circuit current density (J_SC_), open-circuit voltage (V_OC_), and power conversion efficiencies (η) of 1.2 mA/cm^2^, 0.6 V and 0.144%, respectively. Low FF value and conversion efficiency are due to the high series resistance, which is mainly caused by the resistance of the organic material PbPc and the OAD-InN nanocolumnar material, therefore an objective of our planned future work is to reduce the series resistance of cells. 
